# Electrochemical sensory detection of *Sus scrofa* mtDNA for food adulteration using hybrid ferrocenylnaphthalene diimide intercalator as a hybridization indicator

**DOI:** 10.1039/d0ra03585h

**Published:** 2020-07-22

**Authors:** Norzila Kusnin, Nor Azah Yusof, Jaafar Abdullah, Suriana Sabri, Faruq Mohammad, Shuhaimi Mustafa, Nurul Asyikeen Ab Mutalib, Shinobu Sato, Shigeori Takenaka, Nor Azizah Parmin, Hamad A. Al-Lohedan

**Affiliations:** Institute of Advanced Technology, Universiti Putra Malaysia 43400 Serdang Selangor Malaysia azahy@upm.edu.my norzilakusnin87@gmail.com +60 389466782; Department of Chemistry, Faculty of Science, Universiti Putra Malaysia 43400 Serdang Selangor Malaysia; Department of Microbiology, Faculty of Biotechnology and Biomolecular Sciences, Universiti Putra Malaysia 43400 Serdang Selangor Malaysia; Department of Chemistry, College of Science, King Saud University P.O. Box 2455 Riyadh 11451 Saudi Arabia fmohammad@ksu.edu.sa; Halal Product Research Institute, Universiti Putra Malaysia 43400 Serdang Selangor Malaysia; Department of Applied Chemistry, Kyushu Institute of Technology 1-1 Sensui-cho, Tobata-ku Kitakyushu Fukuoka 804-8550 Japan; Institute of Nano Electronic Engineering, Universiti Malaysia Perlis 01000 Kangar Perlis Malaysia

## Abstract

In this study, an electrochemical DNA biosensor was developed based on the fabrication of silicon nanowires/platinum nanoparticles (SiNWs/PtNPs) on a screen-printed carbon electrode (SPCE) for the detection of *Sus scrofa* mitochondrial DNA (mtDNA) in food utilizing a new hybrid indicator, ferrocenylnaphthalene diimide (FND). The morphology and elemental composition of the SiNWs/PtNPs-modified SPCE was analyzed by field emission scanning electron microscopy (FESEM) combined with energy dispersive X-ray spectroscopy (EDX). Cyclic voltammetry (CV) was used to study the electrical contact between the PtNPs and the screen-printed working electrode through SiNWs, while electrochemical impedance spectroscopy (EIS) was used to measure the charge transfer resistance of the modified electrode. The results clearly showed that the SiNWs/PtNPs were successfully coated onto the electrode and the effective surface area for the SiNWs/PtNPs-modified SPCE was increased 16.8 times as compared with that of the bare SPCE. Differential pulse voltammetry used for the detection of porcine DNA with FND as an intercalator confirmed its specific binding to the double-stranded DNA (dsDNA) sequences. The developed biosensor showed a selective response towards complementary target DNA and was able to distinguish non-complementary and mismatched DNA oligonucleotides. The SiNWs/PtNPs-modified SPCE that was fortified with DNA hybridization demonstrated good linearity in the range of 3 × 10^−9^ M to 3 × 10^−5^ M (*R*^2^ = 0.96) with a detection limit of 2.4 × 10^−9^ M. A cross-reactivity study against various types of meat and processed food showed good reliability for porcine samples.

## Abstract 2

An electrochemical DNA biosensor was developed based on the fabrication of silicon nanowires/platinum nanoparticles on a screen-printed carbon electrode for the detection of *Sus scrofa* mitochondrial DNA in food.

## Graphical abstract

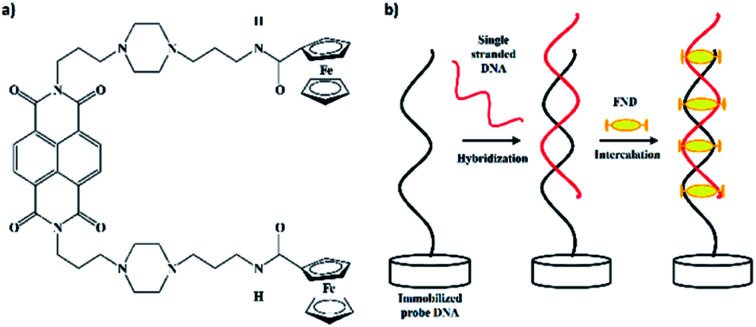

## Introduction

In recent years, there has been increased concern regarding the disclosure of food product ingredients to consumers, and is even mandated by law in third-world countries. Although some countries, such as Canada, Malaysia, Indonesia, Saudi Arabia, *etc.* have very clear policies and regulations that halal food composition must be declared in detail, for some products, the complete identification of food adulteration remains a challenge.^1^ In the past, lard adulteration and the use of pig intestine sausage casing have been major food adulteration issues in Malaysia since they are related to the halal status of food products. Porcine derivatives include pork fat (lard), porcine gelatin, porcine blood plasma and mechanically recovered meat and are usually used by food manufacturers in most countries because of their cheaper price and ready availability.^2^ Hence, in order to supply food and dairy products that are free from porcine derivatives or ingredients of steroidal origin, there is a requirement to develop sensitive and rapid methods that can trace even minute compositions. Currently available techniques suffer from the limitations of time consumption, high analysis costs, poor sensitivity, well-trained personnel, complex instruments, *etc.*, and so halal certification for all products in the market is becoming difficult. As the halal and non-halal certification of products is crucial for some communities, like Muslims, this authentication is expected to increase product consumption and enhance the market value.^3^

Electrochemical biosensing is one of the strategies that can overcome the limitations of existing porcine DNA detection systems in food as analysis can be conducted using portable devices. The technique benefits from rapid response, high sensitivity and easy operation with minimal sample preparation, and also enables continuous monitoring. The success of DNA biosensors is based on the highly specific biorecognition interface and the transducer (electrode), which provide an electronic signal to the end-user. Moreover, a suitable DNA matrix for DNA probe immobilization is also an important criterion for electrochemical performance, as it will provide a large surface area and increase the sensitivity of the developed sensor so that it is able to reach low detection limits.^4^ In this view, the present work deals with the utilization of silicon nanowires (SiNWs) as DNA probe matrices because of their unique optical, mechanical and electrical properties,^5^ in addition to their high surface to volume ratio,^6^ thereby making them a great choice for the development of ultrasensitive sensors. As they have a small size, in the range of 1 to 100 nm, SiNWs appear as good electron transferring agents and result in a fast detection response.

Platinum (Pt) nanoparticles (NPs) have attracted interest in recent advances of electrochemical biosensors as surface modifiers for screen printed electrode (SPE) modification^7^ due to their large specific area, high conductivity, and good compatibility. PtNPs with small particle sizes can be well distributed on the electrode surface, and thereby enhance the conductivity and electron transfer between the substrate and the transducer.^8^ They can also bind with other materials with several functional groups, such as NH_3_, CN or SH,^9^ making them a good nanomaterial for electrode modification. Table 1 shows the utilization of various nanocomposites combined with PtNPs in the fabrication of electrodes to enhance the performance and sensitivity of the developed sensors.

**Table 1:** Utilization of nanocomposites in the fabrication of electrodes

Nanomaterials	Detection	References
Single walled carbon nanotubes/PtNPs	Hydrogen peroxide (H_2_O_2_)	10
Graphene hybrid nanosheet/PtNPs	Hydrogen peroxide (H_2_O_2_) and trinitrotoluene (TNT)	8
Multi walled carbon nanotubes/PtNPs	Glucose	11
Carbon nanotubes/PtNPs	Sudan I	12
Nafion film/Pt–PdNPs	Formaldehyde	13
Chitosan/PtNPs	Erythromycin	9
Polyaniline–graphene nanosheets/PtNPs	Hydrogen peroxide (H_2_O_2_) and glucose	14

Taking into consideration earlier studies, we hypothesized that a combination of SiNWs and PtNPs (SiNWs/PtNPs) could have potential for low detection limits because of the significant enhancement in current as compared to pure SiNWs (without PtNPs). So, SiNWs decorated with PtNPs in the nanocomposite form were first employed in the electrode modification as the DNA probe immobilization and hybridization matrix. The attractive performance of the combination of these two nanomaterials brings new advantages for the design of electrochemical DNA biosensors in terms of high sensitivity and low detection limits.

**Fig. 1:**
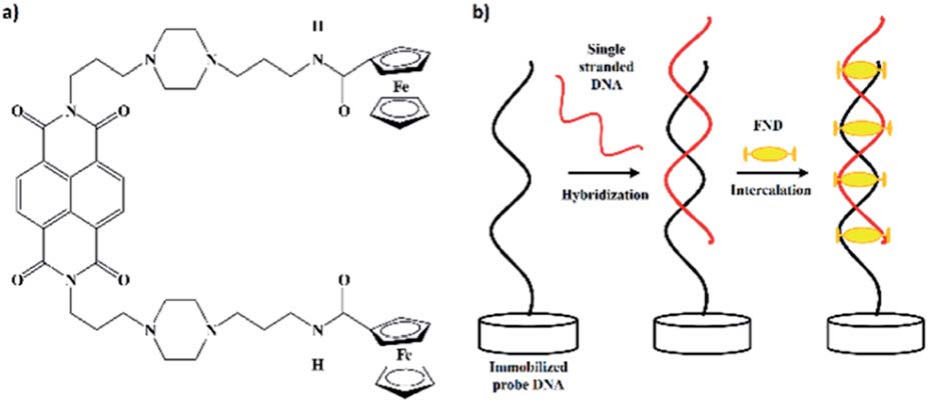
(a) Ferrocenylnaphthalene diimide (FND) structure. (b) Schematic illustration of DNA hybridization with immobilized probe DNA and intercalation of ferrocenylnaphthalene diimide (FND) in between dsDNA.

The effectiveness of the biosensor was further studied using ferrocenyl naphthalene diimide (FND) (Fig. 1a) as a redox label.^15^ In the past, methylene blue (MB) has often been employed in electrochemical detection methods due to its stronger affinity for exposed guanine in single-stranded DNA;^16,17^ however in this work, we have used a new indicator (FND) that specifically binds to double-stranded DNA (dsDNA). The FND molecule binds to dsDNA through a threading intercalation mode,^18,19^ in which the ferrocene molecule is inserted between the duplex and its two peripheral substituents (Fig. 1b). The substituents lie in the major and minor grooves in the complex with a DNA duplex and serve as an anchor to prevent the FND molecule’s dissociation from the dsDNA. This complex is stabilized by van der Waals forces, non-covalent interactions and π stacking, with a slow dissociation rate and high rigidity,^18^ whereas for single-stranded DNA, the FND molecule dissociates readily due to the non-stabilizing interactions between the two peripheral substituents. Thus, this intercalator shows a huge benefit for electrochemical measurements to distinguish between single-stranded and dsDNA.

## Materials and methods

### Reagents

All chemicals were analytical grade and purchased from Sigma-Aldrich (UK). All aqueous solutions were prepared with 18.2 MΩ cm ultra-pure water from Barnstead Nanopure (Thermo Fisher Scientific, USA). Ferrocenyl naphthalene diimide (FND) was obtained from the Takenaka lab, Kyushu Institute of Technology, Japan.

The sequence of porcine oligonucleotides was retrieved from the National Center for Biotechnology Information (NCBI) database with the accession number AJ002189.1. Synthetic oligonucleotides were obtained from Integrated DNA Technologies (IDT) with the following sequences:

Thiolated ssDNA probe: 5′-(HS-(CH_2_)_6_-CTC TAC GTG GAT GTG AAG CAC CGC)-3′

Complementary ssDNA: 5′-GCG GTG CTT CAC ATC CAC GTA GAG-3′

One-base mismatched DNA: 5′-GCG GTG CTT CAG ATC CAC GTA GAG-3′

Three-base mismatched DNA: 5′-GCG GAG CTT CAG ATC CAG GTA GAG-3′

Non-complementary DNA: 5′-CGA TCG TTA ACG CTA ACT GGC CTA-3′

Stock solutions of all oligonucleotides (100 μM) were prepared following the manufacturer’s instructions and were kept frozen at −20 °C for further use.

### Electrochemical measurements

The electrochemical measurements were performed using a μAUTOLAB (III) potentiostat (Eco Chemie, Utrecht, The Netherlands) with a three-electrode system combined as one compartment consisting of a carbon counter electrode, Ag/AgCl reference electrodes and a carbon working electrode. Cyclic voltammetry (CV) and electrochemical impedance spectroscopy (EIS) were carried out for electrochemical characterization of the fabricated electrodes, while differential pulse voltammetry (DPV) was performed for the detection of target DNA sequences. All measurements and analysis were operated using NOVA 1.11 software.

The CV studies were carried out in 1.0 mM [Fe(CN)_6_]^3−/4−^ containing 0.05 M KCl as the supporting electrolyte in the potential range of −0.6 to 0.8 V with a scan rate of 0.1 V s^−1^. The EIS studies were performed under the same supporting electrolyte conditions at 0.20 V. The impedance spectrum was measured over the frequency range from 0.1 kHz to 100 kHz with 10 mV amplitude. The DPV studies were conducted in 50 mM Tris–HCl containing 20 mM NaCl, pH 7.6 as the supporting electrolyte. The DPV scanning potential was monitored in the range of 0.1 to 0.7 V, with 0.025 V modulation amplitude, 0.005 V step potential and an interval time of 0.5 s.

### Synthesis of PtNPs

The PtNPs solution was synthesized based on the previously described method by Bigall *et al.*^20^ with a slight modification. Briefly, a platinum seed solution was prepared by adding 3.6 mL of chloroplatinic acid hexahydrate (H_2_PtCl_6_·6H_2_O) (0.2%) into 46.4 mL boiling deionized water, with vigorous stirring. Then, 1.1 mL of citric acid (0.05%) and sodium citrate (1%) was added, followed by the quick injection of 0.55 mL of a freshly prepared sodium borohydrate (0.08%) solution containing citric acid (0.05%) and sodium citrate (1%). The Pt seeds were obtained when the color of the solution changed from colorless to yellowish and the product was then cooled to room temperature for PtNPs synthesis. PtNPs with diameter 20–30 nm were obtained by adding 1 mL Pt seed solution to 29 mL of deionized water. Then, 0.045 mL of 0.4 M H_2_PtCl_6_·6H_2_O and 0.5 mL of a solution containing sodium citrate (1%) and l-ascorbic acid (1.25%) was added. The temperature was gradually increased over time (∼10 °C min^−1^) until a total reaction time of 30 min. A color change was observed from yellow to black, indicating the formation of PtNPs, and the formed particles were characterized by High-Resolution Transmission Electron Microscopy (HR-TEM) (JEOL JEM-2010HR, JEOL, Japan).

### Fabrication of SiNWs/PtNPs modified electrode

The screen-printed carbon electrode (SPCE) was first activated using 0.1 M NaOH by a CV procedure. 4 μL of SiNWs solution in 2% APTES was added to the working electrode surface and left for 2 h at room temperature. Then, the electrode was baked in an oven at 70 °C for 30 min, after rinsing with 95% ethanol. A 10 μL solution of 5 mM ethanolic 3,3′-dithiodipropionic acid (DTDPA) was drop-casted on the modified SPCE (denoted SPCE/SiNWs) and subjected to 2 h incubation at room temperature. Then, 10 μL of PtNPs suspension was drop-casted at 50 °C onto the SPCE/SiNWs/DTDPA for 15 min, rinsed with deionized water to remove excess PtNPs, and dried with N_2_ gas. Generally, Pt has a high affinity towards sulfur atoms, and the self-assembled layer formation from disulfides is based on the reactions reported by Jiang *et al.* and Shih *et al.* The molecules containing thiol ligands will form dative bonds while interacting with metal surfaces and metal ions. Bidentate thiol ligands were used in this reaction with PtNPs due to the greater bonding adhesion to the electrode surface.R–S–S–R + Pt → 2RSPt

The morphology of the fabricated SPCE (SPCE/SiNWs-PtNPs) was characterized using scanning electron microscopy-energy dispersive X-ray spectroscopy (SEM-EDX).

### Probe DNA immobilization and hybridization

Probe DNA was prepared in TE (Tris EDTA) buffer solution at pH 8.0 and was immobilized on the modified electrode surface by drop-casting 10 μL of 5 μM thiolated ssDNA probe at 4 °C overnight. After immobilization, any unbound thiolated ssDNA probe was removed by rinsing the electrode with TE buffer, and the modified SPCE was denoted SPCE/SiNWs-PtNPs/ssDNA. For the DNA hybridization study, the fabricated SPCE/SiNWs-PtNPs/ssDNA was drop-casted with 10 μL of complementary DNA in TE buffer (pH 8.0) and incubated for 2 h at 40 °C. Then, the electrode was washed with TE buffer to remove any excess complementary DNA and dried with N_2_ gas. This modified SPCE was denoted SPCE/SiNWs-PtNPs/dsDNA.

### Electrochemical DNA detection using FND

**Fig. 2:**
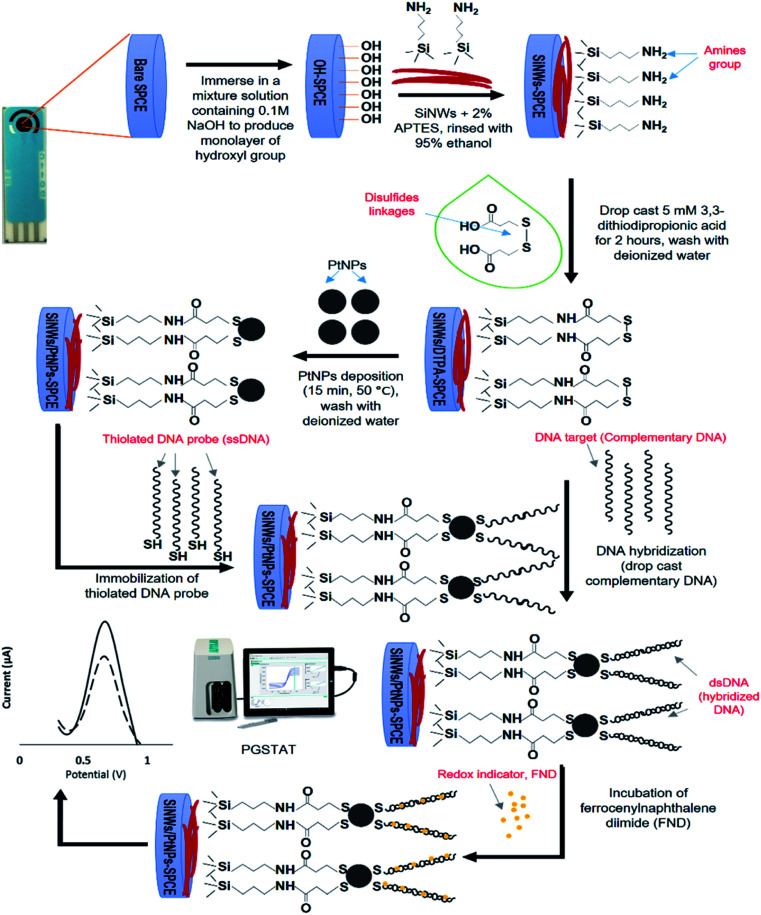
Schematic illustration of the fabrication and DNA hybridization process of SiNWs/PtNPs modified SPCE for electrochemical biosensor.

The success of the DNA hybridization event was measured by the electrochemical signal of the ferrocene molecule from the FND intercalator. Firstly, the hybridized electrode (SPCE/SiNWs-PtNPs/dsDNA) was immersed in 3 mL of the FND solution for 30 minutes. Then, the electrode was carefully rinsed with a mixture of dimethyl sulfoxide (DMSO) and deionized water with a ratio of 1 : 1 to remove excess FND, and lastly dried with N_2_ gas. The oxidation potential of the ferrocene molecule was electrochemically measured using a DPV technique with 50 mM Tris–HCl containing 20 mM NaCl (pH 7.6) as the supporting electrolyte. A schematic illustration of the fabrication and DNA hybridization of the biosensor for SiNWs/PtNPs-modified SPCE is shown in Fig. 2.

### Selectivity and sensitivity

For the selectivity study, the one-base mismatch, three-base mismatch and non-complementary synthetic porcine oligonucleotides were diluted to a concentration of 10 nM each. The fabricated electrode (SPCE/SiNWs-PtNPs/ssDNA) was then introduced to 10 μL of the diluted oligonucleotides. The hybridization event between the immobilized DNA probe and the target is described in the subsection ‘Probe DNA immobilization and hybridization’. In sensitivity studies, different concentrations of complementary target DNA in the range of 3 × 10^−9^ M to 3 × 10^−5^ M were used to measure the detection limit and the effectiveness of the fabricated electrode.

### DNA extraction, PCR, and electrophoresis

Fresh raw meat (pork, lamb, beef) and processed food containing pork was purchased from local markets (Malaysia). The DNA was extracted from all food samples using the DNeasy mericon Food Kit (Qiagen, Germany). The concentration and purity of the extracted DNA samples was obtained using a NanoDrop™ 2000 spectrophotometer (ThermoFisher Scientific, USA). For further use, the DNA samples with purity (*A*_260_/*A*_280_) values ranging from 1.8 to 2.0 were chosen for PCR amplification. The extracted DNA was then amplified in a 50 μL volume reaction mixture containing 1× exTEN 2X PCR master mix, 0.5 μM each of forward and reverse primer, 1 μg DNA template and nuclease-free water (1st Base). The PCR reaction was carried out in a T100™ thermal cycler (Bio-Rad, USA). The PCR cycling conditions involved a single initial denaturation at 95 °C for 5 min, followed by 30 cycles of amplification with denaturation at 95 °C for 30 s, primer annealing at 55 °C for 30 s, and primer extension at 72 °C for 90 s. The final extension step was performed at 72 °C for 7 min. All the amplified PCR products were stored at −20 °C for further use. The amplified PCR product was run in 1.7% (v/v) TBE agarose gel electrophoresis supplemented with 0.1% GelRed (Biotium) to determine the size of the PCR product using a 100 bp DNA ladder (1st Base) as the ladder marker. The gel was then photographed using a UV transilluminator.

## Results and discussion

### Surface morphology of modified SPCE

**Fig. 3:**
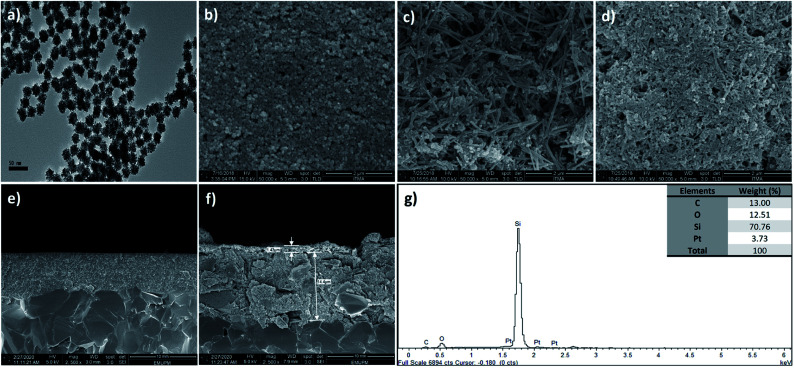
(a) HRTEM of PtNPs. FESEM images for the surface of (b) bare SPCE, (c) SiNWs/SPCE, (d) SiNWs-PtNPs/SPCE with magnification of 50k. FESEM images for the cross-section of (e) bare SPCE and (f) SiNWs-PtNPs/SPCE with magnification of 2.5k, and (g) EDX of SiNWs/PtNPs-modified SPCE.

The size of the synthesized PtNPs was examined using HRTEM with 50k magnification (Fig. 3a); the image shows the uniformity of PtNP distribution and the average nanoparticle size is revealed as 25 nm with 6% standard deviation after analysis using ImageJ software, indicating the presence of monodispersed particles. FESEM was used to examine the changes in the electrode surface morphology before (Fig. 3b) and after drop-casting with SiNWs (Fig. 3c) and SiNWs-PtNPs (Fig. 3d). Fig. 3b shows the FESEM image of bare SPCE, Fig. 3c shows SiNWs uniformly sticking together and forming a bundle due to the van der Waals forces, and Fig. 3d shows PtNPs randomly dispersed on the surface of SiNWs, which indicated that the nanoparticles were successfully deposited on the SPCE/SiNWs surface. Meanwhile, Fig. 3e and f present the FESEM images for the cross-section of bare SPCE and SiNWs-PtNPs with a modified materials thickness of 1.43 μm. The utilization of SiNWs-PtNPs as a DNA immobilization matrix may increase the active surface area of modified SPCE and enable a better detection signal for DNA hybridization. The successful modification of the electrode surface was additionally proven using EDX analysis (Fig. 3e) to figure out the elemental composition of the modified electrodes. The inset of Fig. 3g shows the weight percentage of Si and Pt, which indicates that the electrode was successfully modified with the nanocomposites. There is a high weight percentage of C, O and Si composition due to the modification of the electrode with APTES, which contains silicon, oxygen and carbon.^21^

### Effective surface area of modified SPCE

The performance of the developed DNA biosensor depends on the effective surface area of the modified electrode, and it can be calculated based on the Randles–Sevcik equation:^22^

1 *I*_pa_ = (2.687 × 10^5^)*n*^3/2^*ν*^1/2^*D*^1/2^*AC*

where *I*_pa_ is the oxidation peak current, *n* is the number of electrons transferred in the redox event (*n* = 1), *ν* is the scan rate (V s^−1^), *D* is the diffusion coefficient of [Fe(CN)_6_]^3−/4−^ solution (7.6 × 10^−6^ cm^2^ s^−1^), *A* is the effective surface area of the electrode (cm^2^) and *C* represents the concentration of ferricyanide solution (1.0 mM).

**Fig. 4:**
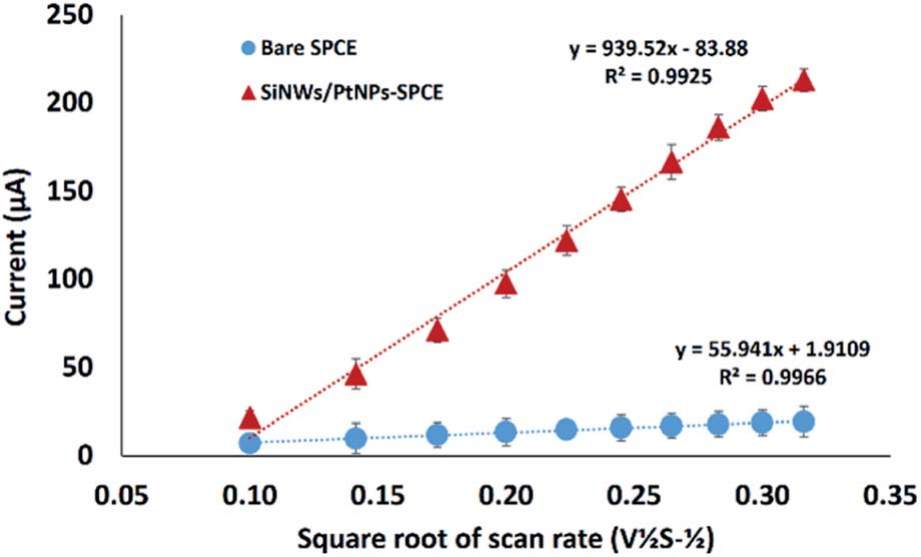
The relationship between the peak potential and square root of scan rate for bare and SiNWs/PtNPs-modified SPCE (*n* = 3).

A CV technique was used to measure the modified and unmodified electrode at different scan rates (10–100 mV s^−1^) with 1.0 mM K_3_[Fe(CN)_6_] containing 0.05 M KCl solution as the supporting electrolyte. The effective surface area for the bare SPCE was calculated as 0.076 cm^2^ and increased to 1.268 cm^2^ for the modified SPCE. The results indicate that modification by the application of SiNWs/PtNPs nanocomposite on the electrode surface increased the effective surface area by about 16.8-fold compared to the unmodified SPCE, which provided advantages for DNA biosensor signal detection. As shown in Fig. 4, the redox peak current of modified SPCE linearly increases with the square root of scan rate (*ν*^1/2^) over the range of 0.1–0.32 V s^−1^, demonstrating that the electrochemical reaction of ferricyanide is controlled by a typical diffusion process. Since the diffusion coefficient and electrolyte concentration are the same, the peak current (*I*_pa_) is mainly affected by the effective surface area (*A*) of the electrode. A previous study by Salinas-Torres *et al.*^23^ showed that the number of electrode electroactive sites that are responsible for the electron transfer correspond to the effective surface area of the electrode. This agreement is supported by Ndlovu *et al.*,^24^ who found that the electroactive site efficiency that can be exposed for the electrocatalytic reaction is highly affected by the effective surface area (*A*).

### The electrochemical characteristics of modified SPCE

**Fig. 5:**
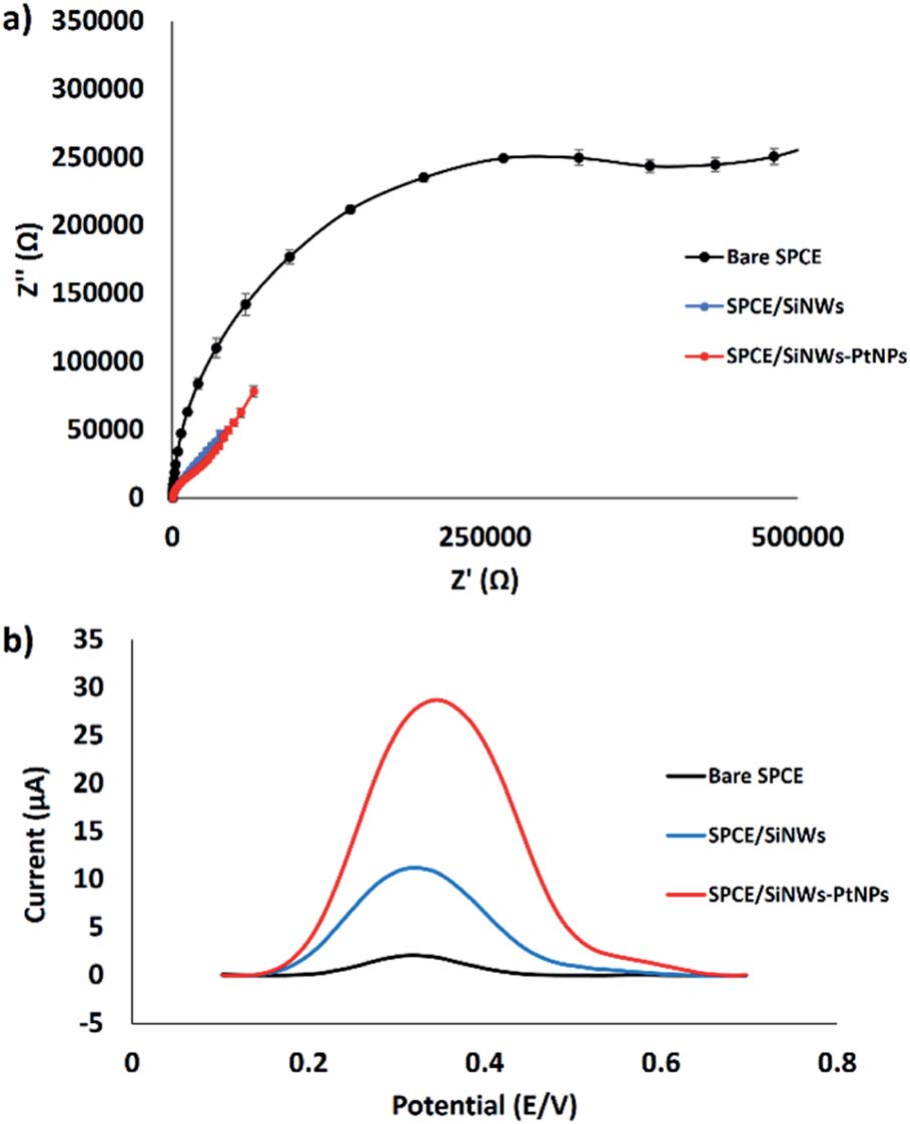
Electrochemical study of the surface modification of SPCE. (a) EIS of modified electrode (*n* = 3). (b) DPV curves of different SPCE modifications after incubation in FND.

The electron transfer resistance (*R*_et_) for the modified electrode was monitored using the EIS technique.^25,26^ The semicircle part shown at high frequency corresponds to the electron transfer resistance (*R*_et_), whereas the semicircle diameter indicates the charge transfer resistance (*R*_ct_). Fig. 5a shows the Nyquist plots of the bare SPCE, SPCE/SiNWs and SPCE/SiNWs-PtNPs in [Fe(CN)_6_]^3−/4−^ containing 0.05 M KCl as the supporting electrolyte. In the presence of nanocomposite, the semicircle portion was observed at low frequencies. Meanwhile, the bare SPCE shows high frequencies, indicating the limitation of electron transfer between the solution and the electrode interface. This agreement corresponds to the result of this work, in which the bare SPCE shows the largest semicircle and the *R*_et_ value obtained is 608 000 Ω. After modification with SiNWs, the value of *R*_et_ decreased slightly to 59 300 Ω, and drastically decreased to 154 Ω when modified with SiNWs-PtNPs. These results suggest excellent electrode modification by SiNWs-PtNPs and are attributed to better electrocatalytic activity. Inversely, the SPCE modified with SiNWs-PtNPs was found to give the best peak enhancement from DPV results after immersion in the FND solution for 30 min (Fig. 5b). The highest DPV peak current at 28.7 μA was acquired when the electrode was modified with SiNWs-PtNPs nanocomposite, as compared with SiNWs alone at 11.2 μA. Fig. 5b shows that the peak current of ferrocene increased from 2.1 μA to 28.7 μA when the nanocomposite (SiNWs-PtNPs) was deposited onto the electrode surface and increased slightly in comparison with bare SPCE in the presence of SiNWs alone. This finding proved that the SiNWs-PtNPs nanocomposite greatly enhanced the electrode conductivity by facilitating electron transfer in the redox process.

### Optimization of experimental conditions

**Fig. 6:**
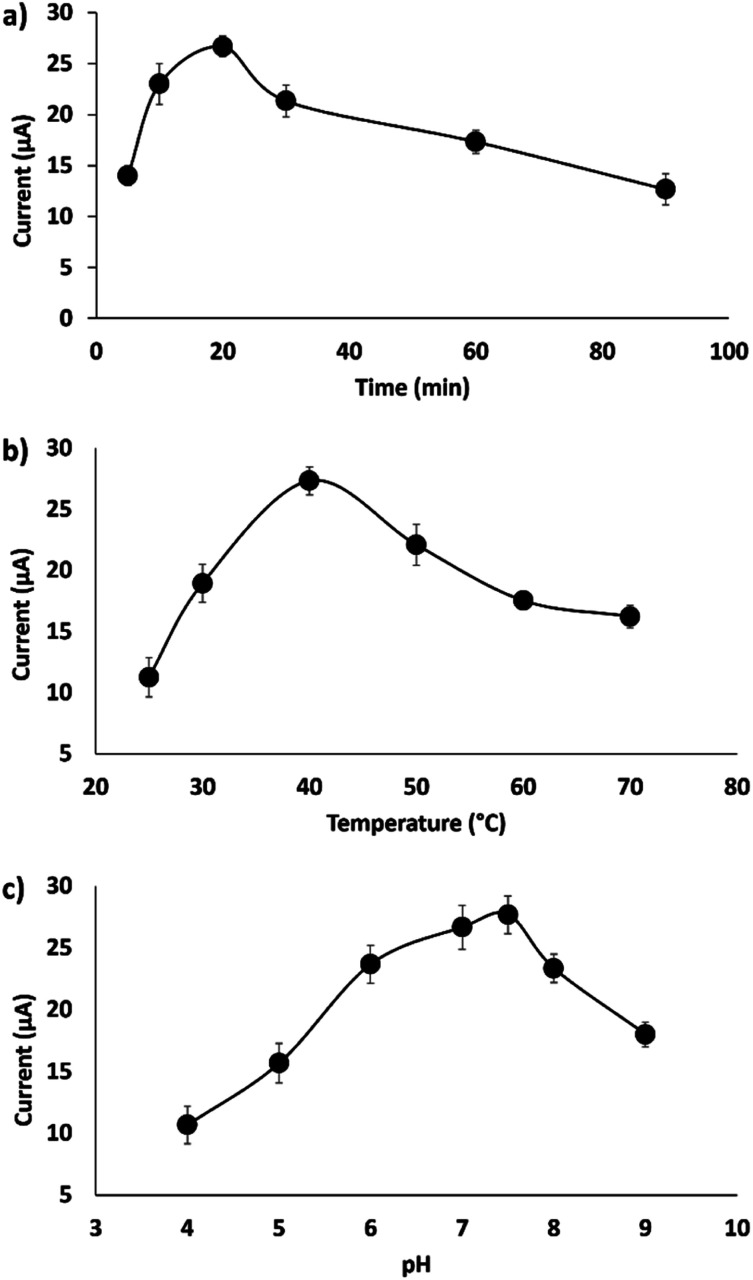
Electrochemical study for the optimization of the hybridization conditions. (a) Optimum hybridization time, (b) optimum hybridization temperature and (c) optimum pH for DNA hybridization (*n* = 3).

The conditions of the hybridization process of the DNA probe with the complementary DNA target were optimized to enhance the hybridization efficiency. Three parameters, including hybridization time, temperature and pH, were studied and optimized to obtain a well-defined DPV peak current. As shown in Fig. 6a, 20 min hybridization time was chosen as the best hybridization condition and is used for the subsequent experiments. In general, an optimum hybridization time is needed to allow the formation of the DNA duplex when the DNA target is exposed to the immobilized DNA probe. Fig. 6a demonstrates that the DNA hybridization efficiency increases from 5 to 20 min and gradually decreases when the time is prolonged up to 90 min. Other work on DNA hybridization by Chen *et al.*^4^ reported that the DNA hybridization efficiency simultaneously increased with hybridization time until a constant state was reached. This indicates that no more hybridization of DNA takes place even when the time is prolonged due to the complete hybridization of the DNA target on the surface of the electrode.

The effect of hybridization temperature is illustrated in Fig. 6b; 40 °C was selected as the optimum hybridization temperature throughout the experiments. This finding is in agreement with the previously documented study by Dutse *et al.*,^27^ which found the optimal hybridization temperature to be between 40 °C and 45 °C. This suggests that a suitable hybridization temperature is important to unfold the immobilized probe DNA and make it available to bind with target DNA, thus increasing the efficiency of DNA hybridization.^28^

The effect of solution pH was measured in the range of pH 4 to 9, as it strongly influences the DNA hybridization rate.^29^ The optimum pH of the solution (Tris–HCl) for DNA hybridization has been identified as 7.6 (Fig. 6c). As shown in Fig. 6c, the DNA hybridization efficiency is enhanced with an increase in pH from 4 to 7.6 and decreases in alkaline solution (pH 8). This result corresponds very well to many previous studies,^29,30^ where it was found that modified gold electrodes using ZnO nanowires showed better detection of breast cancer 1 (BRCA1) oligonucleotides at pH 7. This finding was supported by the agreement that DNA depurination takes place under highly acidic conditions, where adenine and guanine bases are protonated, leading to the breakdown of the N-glycosidic bonds that link the phosphodiester DNA backbone.^31^ An *et al.* demonstrated that the pH level strongly influences the conformation of the DNA structure, where the rate of DNA depurination gradually increases with a decrease in the solution pH. This causes an alteration of the DNA target configuration and interferes in base pairing with the DNA probe, leading to low duplex formation. In alkaline conditions, the double helix DNA will be denatured due to the disruption of hydrogen bonds between DNA strands and will form a single stranded DNA coil.^32^ Therefore, in this study, a neutral pH of 7.6 is chosen as the ideal value for duplex DNA formation.

### Selectivity of the electrochemical DNA biosensor

**Fig. 7:**
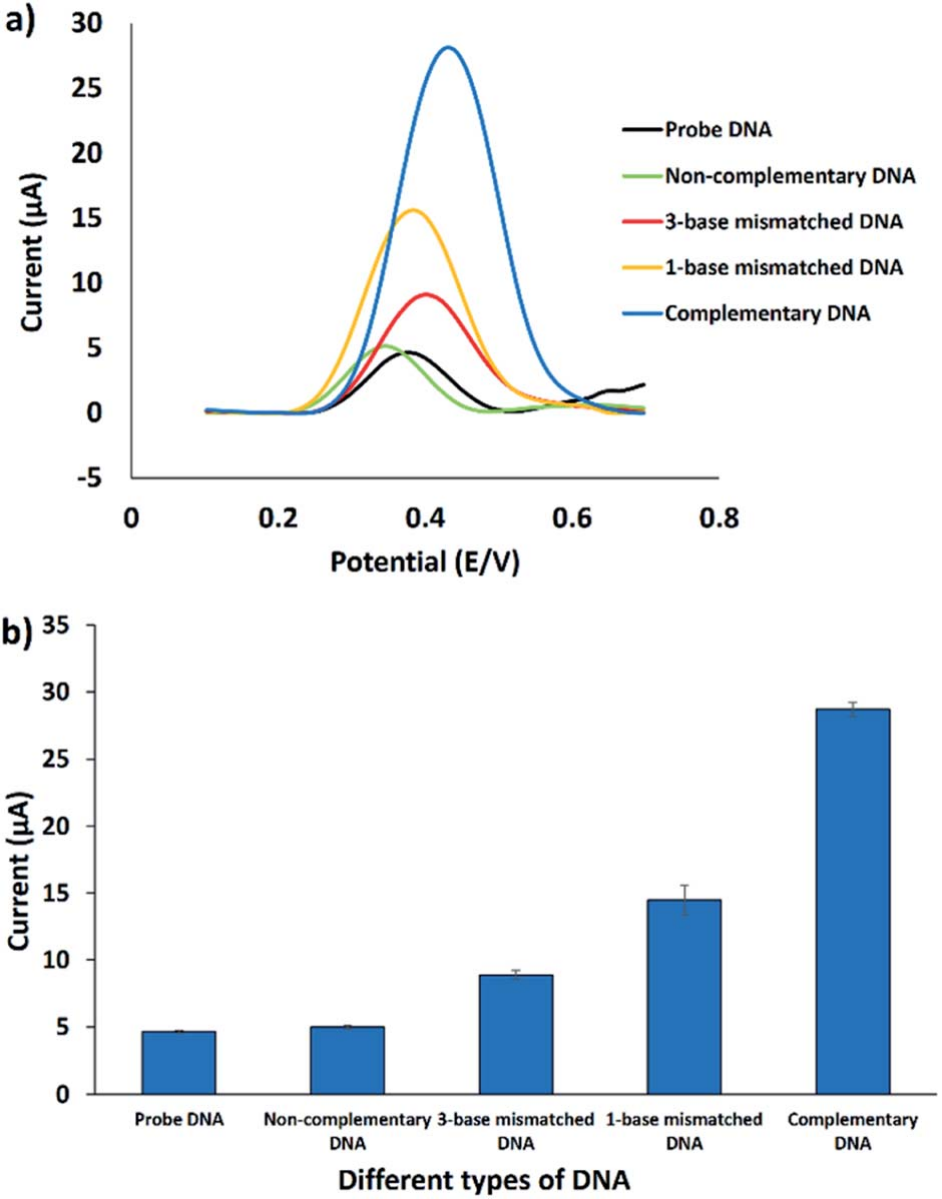
(a) DPV voltammograms of the FND peak current using different types of DNA oligonucleotides in 50 mM Tris–HCl containing 20 mM NaCl (pH 7.6). (b) Histogram of the same conditions (*n* = 3).

The selectivity of the electrochemical DNA biosensor was investigated by introducing different kinds of target DNA to the immobilized probe DNA. Fig. 7a shows the DPV response towards the immobilized probe DNA, complementary DNA, one-base mismatched DNA, three-base mismatched DNA, and non-complementary DNA oligonucleotides, utilizing FND as a redox indicator. FND binds specifically to the hybridized DNA through threading intercalation mode, leading to the accumulation of FND in dsDNA^33^ and resulting in a higher DPV peak current. As shown in Fig. 7b, the current signal of the DPV is similar for non-complementary DNA and the immobilized DNA probe, indicating that no DNA hybridization occurred. The FND peak currents consistently increased when three-base mismatched DNA and one-base mismatched DNA were employed on the immobilized probe DNA. This trend continued for the complementary target DNA. The highest FND peak current (28.2 μA) was obtained with complementary DNA, followed by 1-base mismatched DNA (15.7 μA), 3-base mismatched DNA (9.1 μA), non-complementary DNA (5.1 μA), and probe DNA (4.6 μA). After hybridization with different kinds of target DNA, the FND peak current increased in the order of DNA probe without target < non-complementary DNA < 3-base mismatched DNA < 1-base mismatched DNA < complementary DNA. This finding revealed that the developed DNA biosensor was able to discriminate between complementary and mismatched target DNA, thus increasing the selectivity of the sensor.

### Sensitivity of the SPCE/SiNWs-PtNPs

**Fig. 8:**
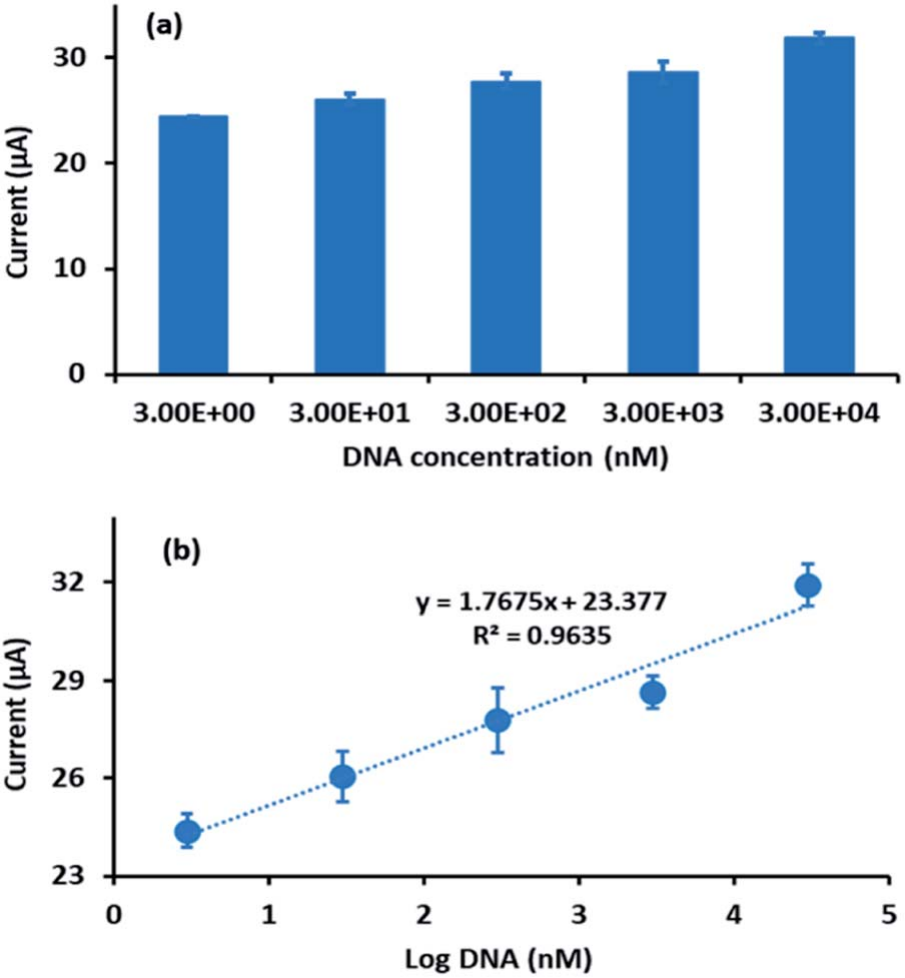
(a) Histogram of DPV peak current of different DNA concentrations. (b) Plot of peak current of FND against logarithmic value of different DNA concentrations (*n* = 3).

The sensitivity of the developed electrochemical DNA biosensor towards different concentrations of complementary DNA target was studied in the range of 3 × 10^−9^ M to 3 × 10^−5^ M. Fig. 8a shows that the DPV peak current increased linearly with an increase of complementary DNA concentration. To determine the linearity of the complementary DNA concentration peak current, a calibration curve (Fig. 8b) was constructed. The curve demonstrated good linearity with a linear regression coefficient of 0.9635. From the experiment, the DPV peak current for the blank (DNA probe) was 5.14 × 10^−6^ A with a standard deviation of 2.46 × 10^−7^ A. This value was used to calculate the differences in the hybridization peak current after immobilized probe DNA was introduced onto the complementary DNA with a range of concentrations from 3 × 10^−9^ M to 3 × 10^−5^ M. Using the 3*σ*/*m* formula^34–37^ (*σ*, standard deviation of blank solution; *m*, slope of the linear curve) (*n* = 3), the limit of detection (LOD) for the developed electrochemical DNA biosensor was calculated as 2.4 × 10^−9^ M.

### Cross-reactivity study of the DNA biosensor against various meat samples

**Fig. 9:**
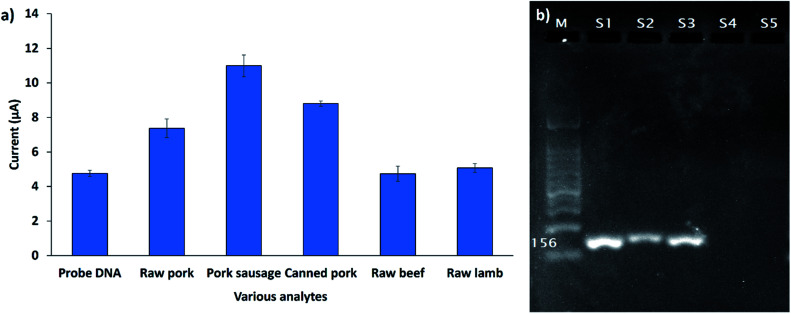
(a) Cross-reactivity study of DNA biosensor against various types of meat samples (*n* = 3). (b) Agarose gel electrophoresis of the PCR products amplified from raw pork, pork sausage, canned pork, raw beef and raw lamb (S1, S2, S3, S4, S5) by the specific 12S rRNA primer. The amplification of 1 μg of sample DNA is shown. M, 100 bp DNA ladder size standard.

The developed biosensor was used for the detection of the 12S rRNA target gene in the genomic DNA extracted from various types of raw meat and processed foods. Fig. 9a demonstrates the DPV response of the developed biosensor (SPCE/SiNWs-PtNPs/ssDNA) after hybridization with different types of genomic DNA extracted from raw pork meat, pork sausage, canned pork, raw beef meat, and raw lamb meat. The DPV response of raw beef meat and raw lamb meat did not change obviously compared to the DNA probe, indicating that no hybridization of DNA occurred. However, the DPV peak current increased when the capture DNA probe was exposed to raw pork meat, pork sausage, and canned pork with a peak current of 7.4 μA, 10.9 μA and 8.8 μA, respectively. This finding demonstrated that the FND provides high-affinity interactions with the dsDNA surface, thus leading to the high FND redox current. Based on this study, it was revealed that the developed biosensor is specific for the *Sus scrofa* mtDNA target sequence. To confirm the above finding, the genomic DNA of all samples was further amplified with the porcine specific primers that were designed based on the 12S rRNA gene sequence (probe DNA). As depicted in Fig. 9b, the primers successfully amplified the genomic DNA extracted from porcine samples (S1, S2, S3) at 156 bp; meanwhile, for the other samples (S4, S5), the band did not appear, indicating that no amplification occurred. This result agrees with the electrochemical detection, thus proving the significant interactions between the DNA probe and *Sus scrofa* target oligonucleotides.

### Reproducibility of the developed DNA biosensor

Reproducibility of the fabricated biosensor is one of the crucial measures to identify the reliability of its working activity. It can be defined as the capability of the developed DNA biosensor to produce equivalent feedback for a repeated experimental setup.^38^ Hence, it is compulsory to examine the reproducibility of the fabricated DNA biosensor for verification of the consistency of this method, since it is an important factor for the biosensor. For the detection of 3 μM *Sus scrofa* mtDNA, a series of five electrodes was arranged. The peak currents within the range of 21.63–22.96 μA were measured from the developed biosensor, as tabulated in Table 2. Based on the measured peak currents for the five electrodes, the relative standard deviation (RSD) can be calculated based on the following equation:

2 RSD = (*σ*/*μ*) × 100

where *σ* is the standard deviation and *μ* is the mean of the measurements. An acceptable RSD of 2.52% was obtained, which indicates that the developed biosensor has a good reproducibility for the detection of low concentration *Sus scrofa* mtDNA.

**Table 2:** Reproducibility of 3 μM of *Sus scrofa* mtDNA

Replicate	Peak current (μA)	Mean (*μ*)	Standard deviation (*σ*)	RSD (%)
1	21.98	22.18	0.56	2.52
2	21.77			
3	22.54			
4	22.96			
5	21.63			

## Conclusion

In conclusion, the application of SiNWs-PtNPs nanocomposite leads to a simple and sensitive electrochemical DNA biosensor for the detection of *Sus scrofa* mtDNA target sequences. The utilization of SiNWs-PtNPs was proven to enhance the active surface area and conductivity of the modified SPCE. This work used a labelling approach using FND as an intercalator, where the sensing signal is based on the accumulation of ferrocene molecules on dsDNA, thus increasing the ability of the developed electrochemical DNA biosensor to differentiate complementary and mismatched sequences. This agreement is due to the special affinity of naphthalene diimide, which intercalates between Watson–Crick base pairs of dsDNA through a threading intercalation technique. The main factor in developing highly sensitive and selective biosensors is the biorecognition element. By coupling effective surface modification with a selective biorecognition element known as probe DNA, we succeeded in discriminating between complementary, non-complementary, one-base mismatch and three-base mismatch target sequences. Based on the findings, higher selectivity was achieved when ferrocene molecules intercalated with hybridized DNA, allowing high electron transfer and thus increasing the signal detection. In principle, this developed electrochemical DNA biosensor might have promising applications in halal food analysis for the detection of porcine DNA target sequences. Overall, we successfully developed a new DNA biosensor related to the halal field which can reliably detect specific *Sus scrofa* mtDNA target sequences from various types of food samples.

## Conflicts of interest

There are no conflicts to declare.

## Supplementary Material
